# Supplementary material for: Immune Checkpoint Inhibitors Combined with Targeted Therapy: The Recent Advances and Future Potentials
Source: Cancers (Basel). 2023 May 22;15(10):2858. doi: 10.3390/cancers15102858 (PMC10216018; doi:10.3390/cancers15102858)
Supplement: Supplementary file 1 [file cancers-15-02858-s001.zip › cancers-2306924-supplementary.pdf]

Supplement Table S1. Ongoing Clinical Trials of Combining ICIs and Targeted Drugs

Disease	Phase	ICIs	Combined drugs	Name /No.	Status
**Angiogenesis inhibitors**					
Early HCC	III	Atezolizumab	Bevacizumab<br>vs. Active surveillance	IMbrave050	Recruiting
Advanced NSCLC	II	Avelumab	Axitinib	NCT03472560	Active, not recruiting
Stage III/IV unresectable melanoma	II	Ipilimumab	Bevacizumab	NCT01950390	Active, not recruiting
**EGFR/HER2 inhibitors**					
Recurrent or metastatic HNSCC	II	Pembrolizumab	Afatinib	ALPHA	Active, not recruiting
Recurrent or metastatic HNSCC	II	Pembrolizumab	Cetuximab	NCT03082534	Active, not recruiting
**PARP inhibitors**					
Stage III/IV non-mucinous EOC	III	Dostarlimab	Niraparib+SOC<br>vs. SOC(Carboplatin+Paclitaxel+Bevacizumab)	NCT03602859	Active, not recruiting
Advanced OC	III	Avelumab	Talazoparib+Paclitaxel+Carboplatin<br>vs. Bevacizumab+Paclitaxel+Carboplatin and<br>Talazoparib+Paclitaxel+Carboplatin	JAVELIN OVARIAN PARP 100	Active, not recruiting
BRCA1/2-deficient OC	I/II	Tremelimumab	Olaparib	NCT02571725	Active, not recruiting
**MAPK/ERK signaling inhibitors**					
Metastatic, recurrent or refractory NSCLC	II	Atezolizumab	Cobimetinib	NCT03600701	Recruiting
MSS metastatic colon	II	Durvalumab	Trametinib	NCT03428126	Recruiting

cancer					
Metastatic radioiodine refractory *BRAF*^{V600} mutant TC	II	Nivolumab	Encorafenib+Binimetinib<br>vs. Encorafenib+Binimetinib	NCT04061980	Recruiting
Advanced or metastatic TNBC	I/II	Pembrolizumab	Binimetinib	NCT03106415	Recruiting
Advanced NSCLC	I	Pembrolizumab	Binimetinib	NCT03991819	Recruiting
**CDK inhibitors**					
ER+ mBC	II	Avelumab	Palbocicli+Endocrine therapy<br>vs. Avelumab+Endocrine therapy	ImmunoADAPT	Recruiting
NSCLC	I	Pembrolizumab	Abemaciclib	NCT02779751	Active, not recruiting
**PI3K/AKT/mTOR signaling pathway inhibitors**					
Advanced UC	II	Nivolumab	Eganelisib<br>vs. Nivolumab	MARIO-275	Active, not recruiting
Metastatic melanoma	I/II	Pembrolizumab	GSK2636771	NCT03131908	Active, not recruiting
HNSCC	I/II	Nivolumab	Copanlisib	NCT03735628	Active, not recruiting
Colon cancer	I/II	Nivolumab	Copanlisib	NCT03711058	Active, not recruiting
**Hormone receptor inhibitors**					
mCRPC	II	Nivolumab	Enzalutamide	NCT03338790	Active, not recruiting
Small cell neuroendocrine PC	II	Cetrelimab	Apalutamide	NCT04926181	Not yet recruiting

HR+, HER2- advanced or metastatic BC	II	Pembrolizumab	Fulvestrant	NCT03393845	Recruiting

HCC: hepatocellular carcinoma; NSCLC: non-small cell lung cancer; HNSCC: head and neck squamous cell carcinoma; OC: ovarian cancer; EOC: epithelial ovarian cancer; TC: thyroid cancer; TNBC: triple-negative breast cancer; BC: breast cancer; mBC: metastatic breast cancer; UC: urothelial carcinoma; mCRPC: metastatic castration-resistant prostate cancer; PC: prostate cancer; MSS: microsatellite stable; ER+: estrogen receptor positive; HR+: hormone receptor positive; HER2-: HER2 negative.
